# Physician involvement in life transition planning: a survey of community-dwelling older adults

**DOI:** 10.1186/s12875-015-0311-0

**Published:** 2015-07-30

**Authors:** Hillary D. Lum, Jared B. Brown, Elizabeth Juarez-Colunga, Marian E. Betz

**Affiliations:** University of Colorado School of Medicine, Aurora, CO USA; Veterans Affairs Eastern Colorado Healthcare System, Denver, CO USA; Colorado School of Public Health, Aurora, CO USA

**Keywords:** Advance care planning, Advance directives, Driving, Geriatrics, Doctor-patient relationships

## Abstract

**Background:**

With many information sources for healthy aging and life transitions, it is unknown whether community-dwelling older adults desire physician involvement in future planning decisions. The study aimed to examine older adults’ experiences and opinions concerning four future planning domains: advance care planning, driving, finances, and housing.

**Methods:**

Adults aged ≥55 years living at a large urban, independent living facility were surveyed with an anonymous, voluntary, paper-based, mailed questionnaire. Survey domains were advance care planning, driving, finances, and housing. For each domain, questions assessed confidence, openness to discussions, information sources, and prior and desired future role of the physician in decision-making by domain. Comparisons across and within domains were determined using Chi-square tests.

**Results:**

The response rate was 56 % (*N* = 457; median age: 75 years; 74 % female). Among advance care planning, driving, and finances, respondents were more confident about what it means to have an advance directive (87 %, 95 % CI 84 − 90 %) than alternative transportation options (46 %, 95 % CI 42 − 51 %). Nearly two-thirds of respondents (64 %, 95 % CI 59 − 68 %) were open to discussing driving cessation, though only one-third (32 %, 95 % CI 28 − 37 %) were open to having a family member determine timing of driving cessation. More individuals (44 %, 95 % CI 39 − 49 %) were open to a physician deciding about when to stop driving. Past discussions with family or friends about advance care planning or finances were common, although past discussions about driving were less common. Respondents reported personal experience and family as key information sources, which were significantly more common than healthcare providers. While prior involvement by physicians in decision-making was rare across all domains, some respondents expressed desire for future physician involvement in all domains, with advance care planning (29 %, 95 % CI 25 − 33 %) and driving safety (24 %, 95 % CI 20 − 28 %) having highest levels of support for future physician involvement.

**Conclusions:**

Some older adults desired more physician involvement in future planning for life transitions, especially related to advance care planning and driving compared to finances and housing. Clinical implications include increased patient-centered care and anticipatory guidance by physicians for aging-related life transitions.

**Electronic supplementary material:**

The online version of this article (doi:10.1186/s12875-015-0311-0) contains supplementary material, which is available to authorized users.

## Background

The geriatric population is growing at an unprecedented rate [1]. By 2050, the United States population over the age of 65 is expected to be 83.7 million, nearly double the current population [2]. With older adults and longer life expectancies, planning for future health and well-being is imperative. Specifically, many adults need to anticipate transitions in common life domains – including advance care planning and future healthcare choices, transportation and driving retirement, financial security, and housing needs. Physicians play a role in counseling related to some of these domains, including advance care planning, older driver safety, and home safety [3–5]. Other life domains, such as financial planning and choice of housing, may have health-related aspects for some patients, although the physician’s role in life transitions related to these domains has not been as well described. Understanding patient preferences for physician involvement in “anticipatory guidance” – analogous to a pediatrician’s role in counseling parents and patients about coming changes [6] – could enhance patient-centered care as healthcare providers in general practice work to help the aging population prepare for life transitions.

Limited prior research has explored patient preferences for physician input in advance care planning and driving safety. One study on patient expectations found that 95 % of patients agreed with the statement, “It is a good idea for doctors to talk to their patients about advance directives” [4]. Despite patients’ desire for input, advance care planning discussions may be limited in clinical settings [7–9], as are discussions about driving safety or anticipated driving retirement [10]. Discussions about driving often occur only after a safety concern, even though earlier, routine discussions might diffuse tensions and avoid some of the negative impacts of driving cessation [11, 12]. A greater understanding is needed of older adults’ desire for physician involvement in future planning related to advance care planning and driving safety.

Even less is known about the older adults’ preferences for physician involvement in financial planning and housing decisions. Future planning in these areas is often minimal, in part from inadequate knowledge and resources. One-third of Americans in their 50’s have not done any financial retirement planning [13]. Housing transitions such as residence in retirement homes increased over the past decade, but it remains unclear what motivates these decisions [13]. Counseling about finances and housing have not traditionally been physicians’ responsibility, although both are affected by overall health status and aging-related changes. It is unknown if patients desire physician input to navigate transitions in these domains.

The goal of this study was to examine older adults’ experiences and opinions concerning future planning related to advance care planning, driving, finances, and housing, including the desired role of physicians in this process. Finances and housing, which are domains that affect all older adults but traditionally have not involved physician guidance, were assessed alongside advance care planning and driving to understand older adults’ desire for “anticipatory guidance” in various domains. Here, we present findings from a survey of community-dwelling older adults related to their desire for physician input in future life transitions that are impacted by aging-related changes in health.

## Methods

### Study design, setting, and participants

We conducted an anonymous, voluntary survey of adults living at a large independent living facility (almost 3500 residents) in an urban area. All respondents were at least aged ≥55 years based on the independent living facility residential policy. Research staff prepared envelopes containing an introduction letter, paper survey, stamped return envelope, and small incentive (two postage stamps). Facility staff distributed sealed survey invitations to all residents in multiple buildings of the independent living facility complex, which was a convenience sample felt to be representative of the entire population. Surveys were mailed back to research staff so facility personnel did not have access to responses. A small number of surveys (*n* = 17) were completed in-person at a driving education seminar at the facility. A total of 821 surveys were distributed. There were no formal exclusion criteria, although the surveys were paper-based surveys in English only. Completed surveys were entered into Research Electronic Data Capture for data management [14]. Survey completion constituted consent, and the Colorado Multiple Institutional Review Board approved this project.

### Survey

Survey questions were pilot-tested in a convenience sample of ten community-dwelling older adults for clarity and content (see Additional file 1). This analysis reports data from the modules on four domains that frequently involve future planning, decision-making, and life transitions (advance care planning, driving, finances, and housing); pre-planned separate analyses on personal readiness for driving cessation, general injury prevention, and views on firearm safety counseling [15] are not included. For each domain, variables included: (a) confidence in the domain; (b) openness to discussions and planning; (c) sources of information (with multiple responses allowed); (d) prior involvement of physician in decision-making; and (e) desired future involvement of physician in decision-making. Additional questions assessed participant demographics, contact with primary care provider, and reasons for moving to the independent living community. All questions concerning confidence, openness, and physician involvement used 5-point Likert scales, with 1 being lowest. For analyses, these scales were collapsed into dichotomous categories (1, 2 and 3; versus 4 and 5).

### Statistical analysis

We described participants’ responses using medians and interquartile ranges (IQR) or proportions and 95 % confidence intervals (CIs). We used Chi-square tests to compare responses across and within domains. All *P*-values were two-tailed, with *P* < 0.05 considered statistically significant. Statistical analyses were performed using Stata 12.1 (StataCorp, College Station, Texas).

## Results

A total of 457 surveys were returned; three were dropped for analysis (one was determined to be a second completion by the same person; an additional two surveys were each completed by a couple rather than an individual). The overall response rate was 56 %. Table 1 shows survey respondent demographic characteristics. Respondents had a median age of 75 and were predominantly retired white women with at least a high school education. Individuals born between 1946 (age 69 in 2015) and 1964 (age 51 in 2015), termed “baby boomers” [16], were 26 % of respondents. There were no individuals under the age of 55 due to the age restriction of the independent living facility. Nearly three-quarters reported a medical visit with their primary care provider within the past six months.

**Table 1:** Sample characteristics (*N* = 457)

Characteristic	*N* (%)	95 % CI
Age (median, IQR)	75 (69-82)	
Age groups		
55−69	122 (27)	23−31
70 and older	315 (69)	65−73
Female	336 (74)	70−76
Race^a^		
White	415 (91)	88−93
Black	24 (5.3)	3.4−7.7
Other/Unknown	14 (3.1)	1.7−5.1
Hispanic ethnicity	9 (2.0)	0.9−3.7
Highest level of education		
≤ High school or equivalent	201 (44)	39−49
Bachelor’s degree	139 (30)	26−35
Master’s or doctoral degree	98 (21)	18−25
Current employment		
Retired/volunteer	345 (75)	71−79
Full-time/part-time	56 (12)	9.4−16
Missing	56 (12)	9.4−16
Relationship status		
Married/living with partner	126 (28)	24−32
Separated/divorced	110 (24)	20−28
Widowed	156 (34)	30−39
Never married/single	52 (12)	8.8−15
Last primary care provider visit		
Within past month	13 (28)	24−33
1−6 months ago	210 (46)	41−51
6−12 months ago	84 (18)	15−22
Over 12 months ago	18 (3.9)	2.4−6.0

Table 1 Legend. Figures may not add to total due to missing data (not shown if <5 %)

^a^Multiple responses allowed

Survey respondents provided perspectives on confidence regarding future planning domains and openness to having family or physician input on advance care planning, driving (i.e. alternative transportation options and driving retirement), and current and future financial options (Table 2). Among advance care planning, driving, and finances, more respondents expressed confidence about what it means to have an advance directive/living will (87 %, 95 % CI 84 − 90 %) than knowledge about alternative transportation options (46 %, 95 % CI 42 − 51 %) or future financial options (53 %, 95 % CI 49 − 58 %). The majority of respondents were also open to discussing advance care planning with others (family, friends, or physicians) and establishing an advance directive/living will. Nearly two-thirds (64 %, 95 % CI 59 − 68 %) of respondents were open to discussing the decision to stop driving, though only one-third (32 %, 95 % CI 28 − 37 %) were open to having a family member make the decision for them. Slightly more (44 %, 95 % CI 39 − 49 %) were willing to have the decision to stop driving be made by a physician. Twelve individuals self-identified as non-drivers; exclusion of their responses did not change the overall responses regarding driving perspectives. Roughly half of participants were open to discussing their current financial situation with someone (53 %, 95 % CI 49 − 58 %).

**Table 2:** Perspectives on family and physician input on future planning experiences (*N* = 457)

Future Planning Domain	*N* (%)	95 % CI
*Advance care planning*		
Confident about what it means to have an “advance directive” or “living will”	398 (87)	84−90
Confident about process of appointing a medical decision-maker	386 (84)	81−88
Open to discussing options for future care with family, friends or physician	397 (87)	83−90
Open to establishing an “advance directive” or “living will”	404 (88)	85−91
Have already written a formal “advance directive” or “living will”	345 (75)	71−79
*Driving*		
Confident in alternative transportation options	211 (46)	42−51
Open to discussing with family how to decide when to stop driving	291 (64)	59−68
Open to having a family member decide time of driving cessation	147 (32)	28−37
Open to having a physician decide time of driving cessation	201 (44)	39−49
*Finances*		
Confident about future financial options	244 (53)	49−58
Confident about current financial situation	294 (64)	60−69
Open to discussing current financial situation with someone	247 (54)	49−59
Open to establishing a financial plan with someone	226 (49)	45−54

**Fig. 1:**
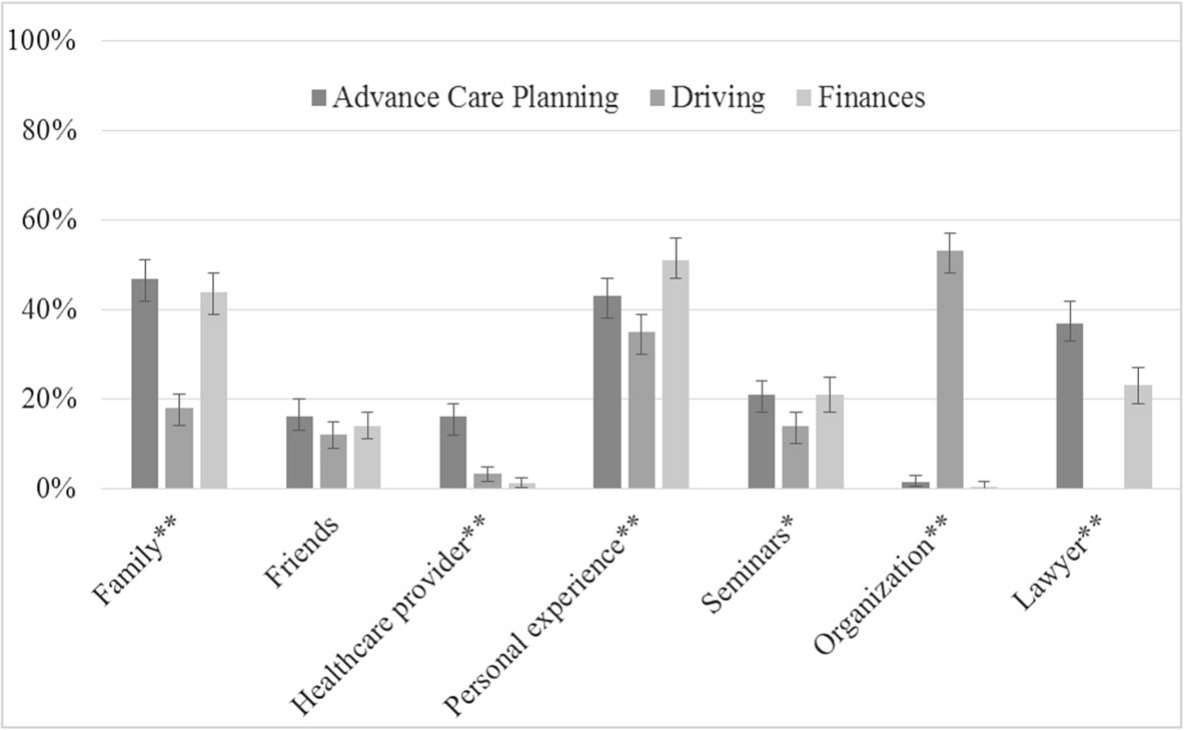
Sources of information for advice, by domain (*N* = 457). More than one response allowed; “lawyer” not available for driving question. Bars represent 95 % Confidence Intervals. **P < 0.001; *P < 0.01 under Chi-square (or *t*-test for lawyer)

When asked about whether they had previously discussed the future planning domains with family or friends, many respondents reported talking about advance care planning (80 %, 95 % CI 76 − 83 %) or financial issues (75 %, 95 % CI 70 − 79 %) compared to fewer who had talked about driving with others (19 %, 95 % CI 16 − 23 %). Individuals reported multiple sources of information for advice on advance care planning, driving safety, and finances (Fig. 1). Personal experience, family, and lawyers were the most common sources for advance care planning information, all significantly greater than physicians (Table 3). Professional organizations, such as AARP (formerly the American Association of Retired Persons), and personal experience were the most common sources of education about driving safety for older adults. Only 3.3 % of respondents identified physicians as a major source of information on driving. As expected, personal experience, financial advisors, and family were the most common sources of information for financial advice. Regarding the housing domain and prior involvement by others in the decision to move to the independent living facility, reported involvement was greatest for family (39 %) and friends (17 %), while only two respondents reported physician involvement.

**Table 3:** Past discussions and sources of information, by domain (*N* = 457)

	Advance care planning		Driving		Finances		*P* value
	N (%)	95 % CI	N (%)	95 % CI	N (%)	95 % CI	
Past discussions with family or friends^a^	364 (80)	76−83	87 (19)	16−23	341 (75)	70−79	0.000
Primary information sources^b^							
Family	214 (47)	42−51	81 (18)	14−21	200 (44)	39−48	0.000
Friends	75 (16)	13−20	55 (12)	9.0−15	63 (14)	11−17	0.160
Healthcare provider	72 (16)	12−19	15 (3.3)	1.6−4.9	6 (1.3)	0.3−2.4	0.000
Personal experience	196 (43)	38−47	159 (35)	30−39	234 (51)	47−56	0.000
Seminars	94 (21)	17−24	62 (14)	10−17	97 (21)	17−25	0.004
Medical insurance provider	NA	NA	9 (2.0)	0.7−3.2	NA	NA	NA
Automobile insurance provider	NA	NA	75 (16)	13−20	NA	NA	NA
Lawyers	171 (37)	33−42	NA	NA	104 (23)	19−27	0.000
Financial advisor	NA	NA	NA	NA	207 (45)	41−50	NA
Organizations (e.g., AARP)	7 (1.5)	0.6−3.1	242 (53)	48−57	2 (0.4)	0.1−1.6	0.000
Media/publications	5 (1.1)	0.4−2.5	6 (1.3)	0.3−2.4	16 (3.5)	2.0−5.6	0.015
Other	9 (2.0)	0.9−3.7	6 (1.3)	0.3−2.4	11 (2.4)	1.0−3.8	0.475

Table 3 Legend**.** Numbers may not add to total due to missing data (not shown if <5 %). *P* values calculated using Chi Square

^a^For advance care planning domain, includes past discussions with family, friends, or physicians

^b^Multiple responses allowed. NA: response not available for this domain

**Fig. 2:**
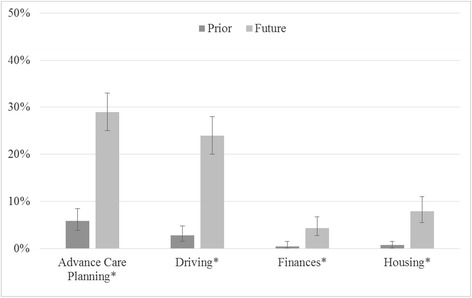
Prior and desired future involvement of physician in decision-making, by domain (*N* = 457). Bars represent 95 % Confidence Intervals. *P < 0.001 under Chi-square within and across categories

Individuals were asked about prior physician involvement in decision-making for each domain, as well as desire for future physician involvement. In all domains, prior physician involvement was very low but desire for future involvement was higher (Fig. 2). As shown in Table 4, the desire for future physician involvement in decision-making was highest related to advance care planning (29 %, 95 % CI 25 − 33) and driving safety (24 %, 95 % CI 20 − 28). Even though some individuals desired future physician involvement in these domains, the majority of respondents (65 − 85 % across the 4 domains) did not have desire for future physician input. Only 11 (2.4 %) people wanted physician involvement in all four domains, while 109 (24 %) of respondents did not want physician involvement in any domain (data not shown), with no differences by age (younger “baby boomer” generation vs older adults) or by gender.

**Table 4:** Prior and desired future involvement of physician in decision-making, by domain (*N* = 457)

Physician Involvement	Advance care planning	Driving	Finances	Housing	*P* value^a^
	N (%) 95 % CI	N (%) 95 % CI	N (%) 95 % CI	N (%) 95 % CI	
Prior involvement	27 (5.9)	13 (2.8)	2 (0.4)	3 (0.7)	0.000
	3.9−8.5	1.5−4.8	0.1−1.5	0.1−1.6	
No prior involvement	410 (90)	418 (91)	426 (93)	421 (92)	
	87−92	89−94	91−95	90−95	
Missing	20 (4.4)	26 (5.7)	29 (6.4)	33 (7.2)	
	2.7−6.7	3.7−8.2	4.2−8.8	4.3−9.0	
Desired future involvement	131 (29)	108 (24)	20 (4.4)	36 (7.9)	0.000
	25−33	20−28	2.7−6.7	5.5−11	
No desire for future involvement	295 (65)	311 (68)	387 (85)	373 (82)	
	60−69	64−72	81−88	78−85	
Missing	31 (6.8)	38 (8.3)	50 (11)	48 (11)	
	4.7−9.5	6.0−11	8.2−14	7.7−13	

Table 4 Legend. Report of physician involvement as “prior involvement” based on rating of 4 or 5 vs “no prior involvement” based on ratings of 1−3. Preference for future involvement as “desired future involvement” based on rating of 4 or 5 vs “no desire for future involvement” based on ratings of 1−3

^a^Chi-square

## Discussion

Some community-dwelling older adults are open to future planning related to advance care planning, driving retirement, and financial planning, and expressed a desire for physician input on these decisions. In this population of older adults living in an independent living facility who reported high rates of recent primary care provider visits, more than half of the participants were open to discussing each of the life transition domains with others, with openness to advance care planning discussions being the most common. Respondents reported using a wide variety of sources of information for future planning. One in four respondents specifically desired more physician involvement in advance care planning and driving-related decisions. Desire for physician input in decision-making related to financial issues or housing was much less common. Given that some older adults may not desire physician input in counseling related to life-transitions, it is essential that physicians and other healthcare providers engage in individualized assessment and counseling tailored to the individual’s needs.

In line with a 2014 Institute of Medicine report emphasizing the need for advance care planning structures within healthcare systems [17], our survey of healthy older adults demonstrates that patients desire these interactions as well. Although almost all respondents reported a lack of prior physician involvement in advance care planning, an interesting finding is that three-quarters reported already having an advance directive. This is at the upper range of the previously reported prevalence of advance care planning in the U.S. of 18 to 70 % [18–20]. The high rates of advance directives in this population may contribute to some respondents reporting no desire for future physician involvement related to advance care planning. Given that some respondents still expressed a desire for physician involvement, our findings suggest that individuals may have unmet needs related to advance care planning. There is clearly an opportunity for healthcare providers, other healthcare team members, and healthcare systems to design, improve, and implement effective patient-centered discussions related to advance care planning.

Similar to advance care planning, respondents also desired more physician involvement in decision-making related to driving safety and driving retirement as compared to their prior experience with physician involvement. Consistent with prior work [21, 22], individuals were open to talking with someone about driving cessation but did not want to have someone else decide for them. If individuals were going to accept input from someone regarding safe driving practices and the potential need to stop, respondents in this study were more open to their physician deciding than a family member. This finding supports physicians providing consistent though individualized counseling, shared decision-making, and support to older adults and families related to planning for driving retirement and identifying alternative transportation options [3, 11]. Given that respondents live in an independent living facility, some may have already decided to stop driving. While the survey did not specifically ask about driving status, 12 people self-identified as non-drivers, and excluding these respondents from the analysis did not change the percentage of missing responses on driving questions.

Although the physician role is important, older adults draw upon diverse sources of information for advice including personal experience, family, and community-based resources. Physicians can conduct a brief needs assessment, provide targeted counseling, and connect patients to helpful resources related to these life transitions domains. To ease survey completion, this survey did not explore patient perspectives on counseling, shared decision-making, or resources from other members of the healthcare team (i.e. social workers, nurses, case managers, etc.). Future work should explore individuals’ openness to input from social workers or other health care professionals on these life transitions domains, including as part of contemporary patient-centered medical home or other team-based clinical models. Brief patient-centered educational resources to link to local or accessible quality resources could be further incorporated.

This study is unique in concurrently exploring experiences and opinions regarding physician involvement in multiple aspects of future life planning. Perhaps not surprisingly, finances and housing are life transition domains that involve future planning but were not identified as domains for physician involvement. Advance care planning and driving, on the other hand, were identified as domains in which older adults desired greater physician involvement. This survey did not explore specific aspects of finances or housing, such as medical insurance or home safety issues, where physicians may play a role. The questions related to housing decisions were limited given the context of all respondents living in an independent living facility that they had already chosen to reside in. Future research could explore potential mechanisms of integrating financial or housing-related counseling services into the healthcare setting and vice versa. Such models move beyond patient-centered models to a broader person-focused approach.

This study has limitations. Given that the survey was conducted at a single independent living facility in an urban area, future assessments should include a broader sampling frame, including older adults living in other community settings including private homes. Survey respondents were 74 % female and 91 % white, which are somewhat higher percentages than in the general US population over 65 years of age (57 % female and 86 % white, based on 2010 US Census data). As a survey, this study is subject to respondent bias and the sample size was too small for detailed subanalyses by demographic groups. Additionally, this survey design did not allow us to collect demographic characteristics of the non-responders to compare to the responders. Some questions had higher levels of missing data (up to 11 %). Specifically, for questions exploring confidence or openness, the range of missing data was 3 − 4 % for advance care planning, 6 − 11 % for driving, and 4 − 9 % for financial issues. Some unanswered questions may have been because respondents felt the question(s) did not apply (i.e. had already completed advance care planning or had already stopped driving). Additionally, as a self-report written survey that potential respondents were asked to complete without assistance, older adults with cognitive, vision, or other health impairment would be less likely to complete the survey and/or potentially have a greater number of missing responses. Taken together, this study likely reports a conservative estimate of older adults with high levels of confidence or openness.

## Conclusion

In conclusion, this survey describes older adults’ confidence in and openness to physician discussions related to future planning for life transitions. The main clinical implication of this study is that some community-dwelling older adults are open and desire to discuss future planning related to advance care planning and driving retirement with their physicians. The challenge and opportunity within the competing demands of providing patient-centered healthcare in primary care is to identify the patient’s priorities, which may include engaging in discussions to promote healthy aging through “anticipatory guidance” related to advance care planning and driving transitions.

## Additional file

Additional file 1:
**Survey instrument.** Survey questionnaire for older adults to understand experiences and opinions related to future planning for life transition domains. (PDF 63 kb)

## Abbreviations

CI: Confidence intervals
IQR: Interquartile range
US: United States
