# Supplementary material for: Physician involvement in life transition planning: a survey of community-dwelling older adults
Source: BMC Fam Pract. 2015 Jul 30;16:92. doi: 10.1186/s12875-015-0311-0 (PMC4518510; doi:10.1186/s12875-015-0311-0)
Supplement: Additional file 1: — Survey instrument. Survey questionnaire for older adults to understand experiences and opinions related to future planning for life transition domains. (PDF 63 kb) [file 12875_2015_311_MOESM1_ESM.pdf]

Additional file 1. Survey Instrument

Thank you for completing this anonymous survey. Your participation is completely voluntary. You may skip any questions you do not want to answer.

DRIVING

The following questions are about your thoughts and preparation surrounding **driving** decisions.

	Not at all ⟵				⟶ Very
How **confident** are you in your transportation options (besides driving yourself)?	1	2	3	4	5
How **open** are you to:					
Discussing with your family how you will decide when to stop driving?	1	2	3	4	5
Having a family member decide when you should stop driving?	1	2	3	4	5
Having a physician decide when you should stop driving?	1	2	3	4	5

Has a family member or friend talked with you about your driving (about safety concerns or future plans to stop driving)? ☐ Yes ☐ No

Where have you learned about driving safety for older adults? (Check all that apply)

- ☐ Family
- ☐ Friends
- ☐ Physician or other healthcare provider
- ☐ Medical insurance providers
- ☐ Automobile insurance providers
- ☐ AARP
- ☐ Personal experience
- ☐ Seminars
- ☐ Other: ______________________
- ☐ I haven’t learned about it

ADVANCE CARE PLANNING

The following questions are about your thoughts and preparation surrounding the kinds of **medical care** you want at the end of life.

	Not at all ⟷				Very
How **confident** are you about:					
What it means to have an "advance directive", also known as a "living will"?	1	2	3	4	5
The process of appointing a medical decision maker, like a durable medical power of attorney?	1	2	3	4	5
How **open** are you to:					
Discussing your options for future medical care with your family, friends, or physician?	1	2	3	4	5
Establishing an "advance medical directive" or "living will"?	1	2	3	4	5

Have you already discussed your values and preferences for future medical care with your family, friends, or physician? ☐ Yes ☐ No

Have you already written a formal "advance medical directive", also known as a "living will"? ☐ Yes ☐ No

Who has provided the majority of your advance care planning advice so far? (Check all that apply)

- ☐ Family
- ☐ Friends
- ☐ Physician or other healthcare provider
- ☐ Attorney/Lawyer
- ☐ Seminars
- ☐ Personal experience
- ☐ Other: _________________

FINANCES

The following questions are about your thoughts and preparation surrounding future **financial** decisions.

	Not at all ⟵				⟶ Very
How **confident** are you about:					
Your financial options in the future?	1	2	3	4	5
Your current financial situation?	1	2	3	4	5
How **open** are you to:					
Discussing your current financial situation with someone?	1	2	3	4	5
Establishing a financial plan with someone?	1	2	3	4	5

Have you already talked about your finances with your family or friends? ☐ Yes ☐ No

Who has provided the majority of your financial advice so far? (Check all that apply)

- ☐ Family
- ☐ Friends
- ☐ Physician or other healthcare provider
- ☐ Financial advisor
- ☐ Attorney/Lawyer
- ☐ Seminars
- ☐ Personal experience
- ☐ Other: ____________________

HOUSING

The following questions are about your thoughts and preparation surrounding **housing** decisions.

How many years have you been living at Windsor Gardens? ______________________

Who helped you decide to move to Windsor Gardens?

	Not Involved ◄				► Very Involved
Family members	1	2	3	4	5
Friends	1	2	3	4	5
Physician/healthcare provider	1	2	3	4	5
Other: __________________	1	2	3	4	5

How much did these **factors** influence your decision to move to Windsor Gardens?

	No Influence ◄				► Strong Influence
Financial concerns	1	2	3	4	5
Health concerns	1	2	3	4	5
Convenience	1	2	3	4	5
Desire for community environment	1	2	3	4	5
Other: ___________________	1	2	3	4	5

ROLE OF THE PHYSICIAN

The following questions are about what you think about the role of your physician (or other primary care provider, like a nurse practitioner or physician assistant).

In the **past**, how involved has your physician been in helping you make decisions about these topics?

	Not Involved ←			→ Very Involved	
Driving	1	2	3	4	5
Advance Care Planning	1	2	3	4	5
Finances	1	2	3	4	5
Housing	1	2	3	4	5

In the **future**, how involved would you like your physician to be in helping you make decisions about these topics?

	Not Involved ←			→ Very Involved	
Driving	1	2	3	4	5
Advance Care Planning	1	2	3	4	5
Finances	1	2	3	4	5
Housing	1	2	3	4	5

When did you last visit your physician or other primary care provider?

- ☐ Within the past month
- ☐ 1 to 6 months ago
- ☐ 6 to 12 months ago
- ☐ Over 12 months ago

PERSONAL INFORMATION

Age:_______ years

Gender ☐ Male ☐ Female

Race (Check all that apply)

- ☐ White
- ☐ African American
- ☐ Asian
- ☐ American Indian/Alaskan Native
- ☐ Pacific Islander
- ☐ Unknown
- ☐ Other:______________________

Ethnicity

- ☐ Hispanic
- ☐ Non-Hispanic

Current employment

- ☐ Full-time employment
- ☐ Part-time employment
- ☐ Volunteer work (unpaid)
- ☐ Retired: ___________ years ago

Marital Status

- ☐ Married
- ☐ Not married, living with a partner
- ☐ Separated or divorced
- ☐ Widowed
- ☐ Never married, single

Highest degree/level of education

- ☐ Less than high school
- ☐ High school diploma or GED
- ☐ Bachelor's degree
- ☐ Master's degree
- ☐ Doctoral degree (MD, JD, PhD)

Thank you for your participation, time and feedback!
Please feel free to leave any comments below.

____

____

____

____

____

____
